# High-Sensitive Detection and Quantitative Analysis of Thyroid-Stimulating Hormone Using Gold-Nanoshell-Based Lateral Flow Immunoassay Device

**DOI:** 10.3390/bios12030182

**Published:** 2022-03-19

**Authors:** Santosh Kumar Bikkarolla, Sara E. McNamee, Paul Vance, James McLaughlin

**Affiliations:** 1 School of Engineering, Engineering Research Institute, University of Ulster, Newtownabbey BT37 0QB, UK; se.mcnamee@ulster.ac.uk; 2 Randox Laboratories Ltd., 55 Diamond Road, Crumlin, County Antrim BT29 4QY, UK; paul.vancee@gmail.com

**Keywords:** lateral flow immunoassay, gold nanoshells, gold nanoparticles, thyroid-stimulating hormone, human serum, human plasma

## Abstract

Au nanoparticles (AuNPs) have been used as signal reporters in colorimetric lateral flow immunoassays (LFAs) for decades. However, it remains a major challenge to significantly improve the detection sensitivity of traditional LFAs due to the low brightness of AuNPs. As an alternative approach, we overcome this problem by utilizing 150 nm gold nanoshells (AuNSs) that were engineered by coating low-density silica nanoparticles with a thin layer of gold. AuNSs are dark green, have 14 times larger surface area, and are approximately 35 times brighter compared to AuNPs. In this study, we used detection of thyroid-stimulating hormone (TSH) in a proof-of-concept assay. The limit of detection (LOD) with AuNS-based LFA was 0.16 µIU/mL, which is 26 times more sensitive than the conventional colorimetric LFA that utilizes AuNP as a label. The dynamic range of the calibration curve was 0.16–9.5 µIU/mL, making it possible to diagnose both hyperthyroidism (<0.5 µIU/mL) and hypothyroidism (>5 µIU/mL) using AuNS-based LFA. Thus, the developed device has a strong potential for early screening and diagnosis of diseases related to the thyroid hormone.

## 1. Introduction

Thyroid-stimulating hormone (TSH) is a glycoprotein that is secreted by thyrotroph cells in the anterior pituitary gland. In turn, it stimulates the biosynthesis and secretion of thyroid hormones, including triiodothyronine (T3) and thyroxine (T4), into the blood [1,2,3]. TSH plays an important role in human development by regulating the body’s metabolism, temperature, weight, and cholesterol [4,5,6]. If a thyroid-related disease is left untreated, it can lead to serious symptoms, such as weight loss or gain, infertility, muscle weakness, osteoporosis, and a range of autoimmune diseases [7,8]. Thus, testing and monitoring to maintain TSH levels in the normal range is important in patients with thyroid-related diseases.

Thyroid-related diseases are the most common endocrine disorders that is diagnosed in 5–13% of the adult population [9,10,11]. Over the last few decades, direct measurement of TSH levels from finger-prick blood samples or blood from the vein is considered as the first-line test for the initial evaluation of thyroid gland function. The normal TSH concentration for a healthy individual is in the range of 0.5 to 5 µIU/mL [12,13]. A serum TSH concentration below the lower limit of the normal range indicates hyperthyroidism [12,13]. In the case of subclinical hypothyroidism, TSH levels are in the range of 5 to 10 µIU/mL, and TSH levels above 10 µIU/mL is considered as thyroid failure [14,15]. Normally, TSH is measured by immunoassays that employ various labels, such as chemi- or bioluminescent molecules, fluorophores, and enzymes, and the signal is optically detected [2,16,17]. Although most of these assays show LOD < 0.01 µIU/mL, they commonly require expensive equipment, qualified technicians, and laboratory conditions.

Point-of-care testing (POCT) is defined as medical diagnostic testing performed at the time and place of patient care to reduce the sample-to-answer time and thus enable faster treatment. Lateral flow immunoassays (LFAs) are considered point-of-care sensors due to their simplicity, low cost, and ease of use and because they generate colored signals that can be read by the naked eye [18,19,20]. As a result, LFAs are used in various industries, such as diagnostics [21,22], drug testing [23], agriculture [24], food safety analysis [25,26,27], and water pollution detection [28]. Their success also lies in their simple design, which has not changed significantly since their first use as pregnancy tests [29]. Although LFAs rely on passive forces to move the sample along the device to generate test lines, their fabrication is tedious due to several optimization steps [30]. To provide the end-user with an easy-to-use device, developers need to optimize several parameters to obtain optimum performance.

AuNPs are widely used in colorimetric LFAs as signal reporters due to their superior physicochemical properties [31,32]. First, they offer intense color signals due to the optical phenomenon called localized surface plasmon resonance (LSPR) [33,34,35]. Second, they can easily be functionalized with various bioreceptors, such as proteins, antibodies, and DNA, through physisorption or thiol–gold chemistry [36,37,38]. Third, they are highly stable due to their inert nature, enabling them to survive in harsh environments. Moreover, the synthesis of AuNPs is scalable and reproducible in standard chemical laboratories [39]. Over the last few years, several AuNP-based LFAs have been commercialized. Being fast and simple are key features of LFAs, but they also come with the drawback of lower sensitivity that does not match laboratory-based sensing techniques. Although AuNP-based LFAs are used in many applications, improving their sensitivity without adding complexity to the device is a challenge [40]. Note that high-sensitivity detection is required in many applications where the concentration of biomarkers is low, such as early detection of cancers and infectious diseases [41,42]. One of the main reasons for the lack of sensitivity of traditional LFAs is the brightness of the 40 nm AuNPs. Despite the ubiquity of colloidal gold nanoparticles, many other labels have been developed to improve the sensitivity of LFAs. For example, different shapes of gold have been explored. Gold nanomaterials of various shapes, including nanorods, nanowires, nanostars, and nanoshells, are commercially available [39]. However, reports of their use as labels in LFA are limited. By comparing thermal contrast and optical contrast, Qin et al. showed that gold nanorods and gold nanoshells may provide higher sensitivity compared to colloidal gold nanoparticles [43]. Yang et al. developed LFAs with gold nanocages and observed 2.5 times improvement in sensitivity compared to gold nanospheres [44]. Labels other than gold, such as platinum nanoparticles [45,46], silver nanoparticles [47], carbon nanoparticles [48], latex microbeads [49], europium microspheres [50], chemiluminescence labels [51], and cellulose nanobeads [52], have also been used to improve the sensitivity of LFA devices. Platinum, which can catalyze the oxidation of chromogenic substrates, such as 3,3′,5,5′-tetramethylbenzidine (TMB) and 4-chloro-1-naphthol/3,3-diaminobenzidine (CN/DAB), can be coated onto gold nanoparticles for signal amplification purposes. The colored mixture generated at the test line due to oxidation of the chromogenic substrate is several orders of magnitude higher than the signal generated by bare AuNPs. Gao et al. coated ultrathin layers (<10 atomic layers) of platinum on Au nanoparticles as a dual-mode label. The low-sensitivity mode used plasmonic AuNP to produce the test line signals, whereas the high-sensitivity mode produced a signal by catalyzing the chromogenic substrate. The performance of the low sensitivity mode was the same as conventional LFA. The high-sensitive mode achieved 100 times improvement in the limit of detection after 5 min incubation with the chromogenic substrate (CN/DAB) [46].

In the market, CIGA Healthcare and Boditech currently provide fluorescence-based LFAs for the detection of TSH in the range of 0.1 to 100 µIU/mL. So far, no colorimetric-based LFAs for the detection of hyper- and hypothyroidism have been reported. In this study, we utilized TSH assay with 150 nm gold nanoshells to obtain a performance in the clinical range with a limit of detection of 0.16 µIU/mL and dynamic range of detection from 0.16 to 9.5 µIU/mL in human serum. In addition, we compared the performance of AuNS-based LFA with AuNP-based LFA for the detection of TSH. The AuNS-based LFA outperformed AuNP-based LFA with enhanced LOD, with nearly 26-fold increase in LOD being observed. The improved performance was due to the higher brightness and larger surface area of gold nanoshells. TSH recovery was assessed with quality controls that contained 50 additional analytes and human plasma samples with TSH levels in the normal range to validate the developed assay.

## 2. Materials and Methods

### 2.1. Materials

Nitrocellulose (NC) membrane roll (UniSart, CN95) of 25 mm width and 100 μm backing was purchased from Sartorius, Göttingen, Germany. Absorbent pad (A238) and glass fiber roll (8951) of 17 mm width were purchased from Kenosha tapes, Amstelveen, The Netherlands. Backing cards of 60 mm width and 0.01” thickness were obtained from Lohmann, Hebron, KY, USA. The monoclonal mouse anti-TSH antibody (10-2428, Fitzgerald) and monoclonal mouse anti-TSH antibody (10-2426, Fitzgerald) were used as capture and detection antibodies, respectively. The monoclonal goat antimouse IgG antibodies (7455507, Lampire Biologicals Laboratories, Pipersville, USA) were used as control-line antibodies. Both TSH calibrators and quality controls (IA 2633) were based on human serum and obtained from Randox Laboratories, Antrim, Northern Ireland. Disease-state plasma of human origin at normal levels of TSH was obtained from Bio-IVT. TSH-depleted serum (36000D) with TSH levels less than 0.1 µIU/mL was obtained from Seqens, Ecully, France. Further information about calibrators, quality controls, and human plasma are shown in the supporting information. Carboxyl-coated gold nanoshells (GSXR150-30M) of 150 nm size and gold nanoparticles (AUXR40-30M) of 40 nm size were obtained from nanoComposix, San Diego, CA, USA. Sulfo-*N*-hydroxysuccinimide (ab145608) was purchased from Abcam, Cambridge, UK. 1-Ethyl-3-(3-dimethylaminopropyl) carbodiimide (EDC, 03449-1 G), phosphate-buffered saline (PBS), 10× Tris buffer solution, bovine serum albumin (BSA), hydroxylamine (467804-10 mL), Tween-20, and desiccants (Z163570) were purchased from Sigma Aldrich, Gillingham, UK.

### 2.2. Instruments

A ZX1010 dispense platform from Biodot (Chichester, UK) was used for deposition of antibodies onto NC membrane and spraying conjugate onto the conjugate pad. A Leelu reader (LUMOS-V3-03) from Lumos Diagnostics (San Diego, CA, USA) was used to analyze the test lines. A lambda 750/650 spectrometer from PerkinElmer (Beaconsfield, UK) was used to collect UV–vis absorption spectra. A CO_2_ laser system (VLS 2.30) from Universal Laser Systems (Scottsdale, AZ, USA) was used to engrave the NC membrane. High-magnification transmission electron microscopy (TEM) images were obtained with a JEOL JEM 2011, Freising, Germany.

### 2.3. Antibody Conjugation onto Gold Nanoshells

The TSH detection antibody was purified using an Amicon filter unit to remove any amine-terminated molecules, which can interfere in the conjugation process, and the purified antibodies were suspended in 10 mM potassium phosphate buffer of 7.4 pH. AuNS–TSH antibody conjugate was prepared by following the protocol from nanoComposix with some modifications. To 1 mL of gold nanoshells (20 OD), 8 μL of freshly dissolved 52 mM EDC and 16 μL of freshly dissolved 46 mM sulfo-NHS were added and incubated for 30 min. To remove excess EDC and sulfo-NHS, the reaction mixture was centrifuged at 8000 rpm for 10 min. The supernatant was carefully removed, and the pellet was resuspended in 1 mL of reaction buffer (0.01× PBS, 5 mg/mL of PEG 20K, pH: 7.4) after washing two times with reaction buffer. Next, 12 μL (60 μg) of purified TSH detection antibody was added and incubated for 60 min followed by the addition of 10 μL of hydroxylamine. The nanoshells were washed three times by centrifuging at 6000 rpm for 10 min, removing the supernatant, and resuspending in 1 mL of conjugate diluent (5 mM PBS, 0.5% BSA, and 0.5% Tween-20). When spraying the conjugate onto glass fiber, 2% sucrose was added to the conjugate diluent.

### 2.4. Antibody Conjugation onto Gold Nanoparticles

To 1 mL of gold nanoparticles (20 OD), 20 μL of freshly dissolved 52 mM EDC and 40 μL of freshly dissolved 46 mM sulfo-NHS were added and incubated for 30 min. To remove excess EDC and sulfo-NHS, the reaction mixture was centrifuged at 8000 rpm for 10 min. The supernatant was carefully removed, and the pellet was resuspended in 1 mL of reaction buffer (5 mM phosphate buffer solution, 5 mg/mL of PEG 20K, pH: 7.4) after washing two times with the reaction buffer. Next, 20 μL (100 μg) of purified TSH detection antibody was added and incubated for 60 min followed by the addition of 10 μL of hydroxylamine. The nanoparticles were washed three times by centrifuging at 8000 rpm for 10 min, removing the supernatant, and resuspending in 1 mL of conjugate diluent (5 mM PBS, 0.5% BSA, and 0.5% Tween-20). When spraying the conjugate onto glass fiber, 2% sucrose was added to the conjugate diluent.

### 2.5. Fabrication of LFDs

TSH capture antibodies and control line antibodies were dispersed in printing buffer (10 mM PBS, 1% sucrose, pH: 7.4) at a concentration of 2 mg/mL. Both the antibodies were dispensed onto the NC membrane using a Biodot (ZX1010) dispense platform at a flow rate of 1 μL/cm to obtain a test line and control line width of 1 mm. The NC membrane was dried in an oven at 37 °C for 60 min. A CO_2_ laser was used to engrave the NC membrane with 1 mm thick lines that were separated by 5 mm for cutting LFA test strips. Gold conjugate was sprayed onto a glass fiber pad at a flow rate of 5 μL/cm and dried in an oven at 37 °C for 120 min. Finally, the NC membrane, conjugate pad, and absorbent pad were manually assembled on a plastic backing card and cut into 5 mm test strips with scissors. The LFA strips were stored in sealed aluminum foil bags with a desiccant to absorb moisture. A digital photograph of the LFD strip is shown in Figure S1.

### 2.6. Specimen Preparation

TSH calibrators and quality controls were prepared by adding 1 and 5 mL of deionized water to lyophilized powders, respectively, followed by one hour of incubation. TSH calibrators ranged from 85.49 to 0.32 µIU/mL in human serum. Quality controls at three different levels, namely high (20 µIU/mL), medium (2 µIU/mL), and low (0.2 µIU/mL), were used to validate the assay. It is worth mentioning that the quality controls contained 50 additional analytes in addition to TSH. Human plasma samples with TSH levels in the normal range were used to check the validity of the assay. Further information about the specimens is given in the supporting information.

### 2.7. Assay Performance

AuNP conjugate was diluted by 4 times with conjugate diluent and sprayed onto the conjugate pad at an application volume of 10 μL/cm, and AuNS conjugate was concentrated by 2 times and sprayed onto the conjugate pad at an application volume of 10 μL/cm. It is worth noting that under the above conditions, the number of AuNP conjugate nanoparticles and AuNS conjugate nanoshells were approximately equal. To each reaction well, 45 µL of the running buffer (10 mM PBS, 2% BSA, and 1% Tween-20) and corresponding volume of TSH specimen were added to obtain the reaction solution. The LFD was immersed into each sample well and allowed to react for 15 min for 10 and 25 µL of TSH samples or 30 min for 50 µL of TSH sample. After the assay time, the LFDs were analyzed using a Lumos colorimetric reader. We calculated the limit of detection (LOD) as Absorption_LOD_ = Absorption_blank_ + 3σ_blank_ (i.e., corresponding value of blank plus three times its standard deviation) [53,54]. Each set of experiments was repeated three times to check the reproducibility.

## 3. Results and Discussion

**Figure 1:**
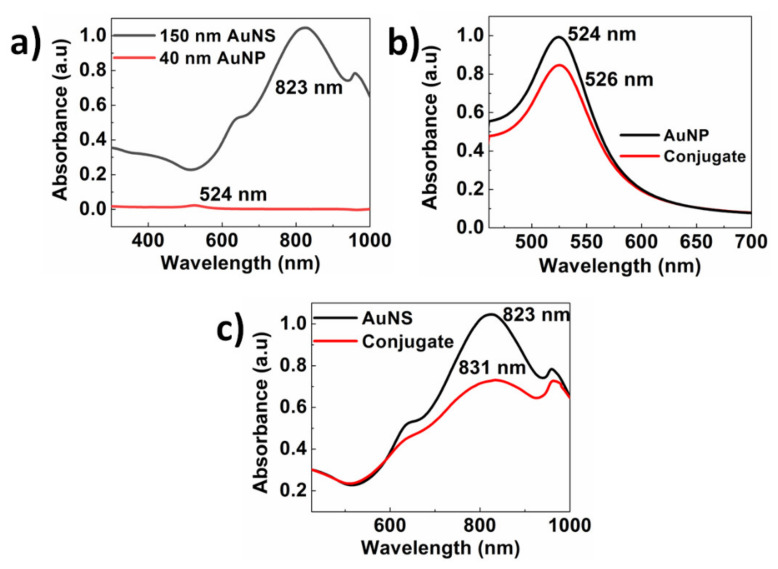
(**a**) UV–vis spectra of carboxyl-coated 150 nm gold nanoshells and 40 nm gold nanoparticles at equal particle number. (**b**) UV–vis spectra of 40 nm gold nanoparticles before and after conjugation. (**c**) UV–vis spectra of 150 nm gold nanoshells before and after conjugation.

UV–vis absorption spectra were collected to investigate the plasmonic properties and stability of AuNPs, AuNSs, AuNP–TSH conjugates, and AuNS–TSH conjugates. All the materials were diluted to 1 OD, and UV–vis spectra were acquired using a quartz cuvette of 300 µL volume and 1 cm path length. The as-obtained UV–vis spectra are shown in Figure 1, and the uncertainty in the plasmonic peak position for all the spectra was less than 1 nm. Figure 1a shows the UV–vis spectra of 40 nm AuNPs and 150 nm AuNSs obtained at equal particle number. In the case of AuNSs, the resonance peak was observed at 823 nm with OD close to 1. In the case of AuNPs, the resonance peak was observed at 526 nm with an OD of 0.022. The LSPR wavelength (i.e., absorption resonance peak) for gold nanoshells was increased due to two reasons. The first reason was because the gold nanoshells were 14 times larger in surface area, which enabled light to interact with a larger electron cloud and resulted in an increase in surface plasmon resonance energy. The second reason was the presence of silica core material, which resulted in a decrease in coulombic restoring force acting on the electron cloud compared to the AuNP and also increased the surface plasmon resonance energy [55,56]. This showed that 150 nm AuNSs were approximately 35 times brighter than 40 nm AuNPs (Figure 1a). For LSPR biosensor applications, refractive index sensitivity (RIS) is one of the critical parameters that show the ultrasensitive nature of the developed device. RIS is defined as the ratio of LSPR wavelength shift to the variation of the refractive index of the surrounding medium [56]. It has been reported that the RIS of gold nanoshells is in the range of 1460–1490 nm/RIU, whereas the RIS of gold nanoparticles is 44 nm/RIU [57,58]. This shows that RIS of gold nanoshells is 33 times higher than gold nanoparticles, which indicates that gold nanoshells are more suitable for plasmonic-based applications compared to gold nanoparticles. It can be observed from Figure 1b that the AuNP–TSH conjugate was prepared with OD of 0.85 and from Figure 1c that the AuNS–TSH conjugate was prepared with OD of 0.73. In both cases, a decrease in OD was observed due to aggregation of particles and loss of material during various stages of the conjugation process.

**Figure 2:**
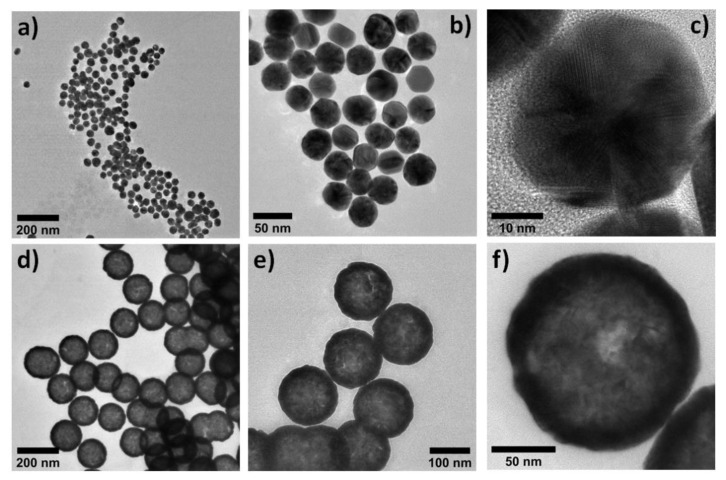
(**a**–**c**) TEM images of 40 nm gold nanoparticles. (**d**–**f**) TEM images of 150 nm gold nanoshells.

In addition, the resonance peak of both the conjugates shifted to the right because of the conjugation of the antibody onto the surface of the reporter nanoparticle. Figure 2a–c shows TEM images of the carboxyl-coated Au nanoparticles with an average size of 40 nm. Figure 2d–f shows TEM images of the carboxyl-functionalized 150 nm Au nanoshells. From Figure 2d, it can be observed that the AuNSs were transparent to high-energy electron beam, indicating the low-density nature of the silica core. It can also be observed that silica nanoparticles of size 120–130 nm were coated with 15–20 nm gold.

Several TSH antibodies were obtained from various suppliers to find suitable antibodies for LFA-based detection. Different configurations (n = 21) regarding detection and capture antibodies were evaluated during the initial antibody screening and characterization. From this initial screening process, Fitzgerald TSH antibodies were identified as promising candidates and were utilized for the remainder of the study. Capture antibodies bind to the intersection region of alpha and beta subunits of TSH antigen, while detection antibodies bind to the beta region of the TSH antigen. By the choice of the antibody pair, we removed the cross-reactivity with LH and FSH alpha subunits. To optimize the TSH assay, AuNS–TSH conjugate was prepared at antibody concentrations of 15, 30, 60, and 120 µg/mL. From this optimization process, 60 µg/mL was found to be suitable for performing the TSH assay. Calibrators were obtained at different concentrations in the form of lyophilized powders, which resulted in the following concentrations: 85.5, 43.9, 23.1, 9.5, 7.8, 5.5, 4.1, 1.0, and 0.32 µIU/mL in human serum after reconstitution with DI water. To prepare even further lower concentrations, the calibrator with 0.32 µIU/mL concentration was diluted with DI water. For blank, TSH-depleted serum (Seqens) with TSH levels lower than 0.1 µIU/mL was used. Furthermore, we performed experiments with calibrators ranging from 85.49 to 0.01 µIU/mL and a blank. We evaluated test line and control line absorptions using a Lumos colorimetric reader.

**Figure 3:**
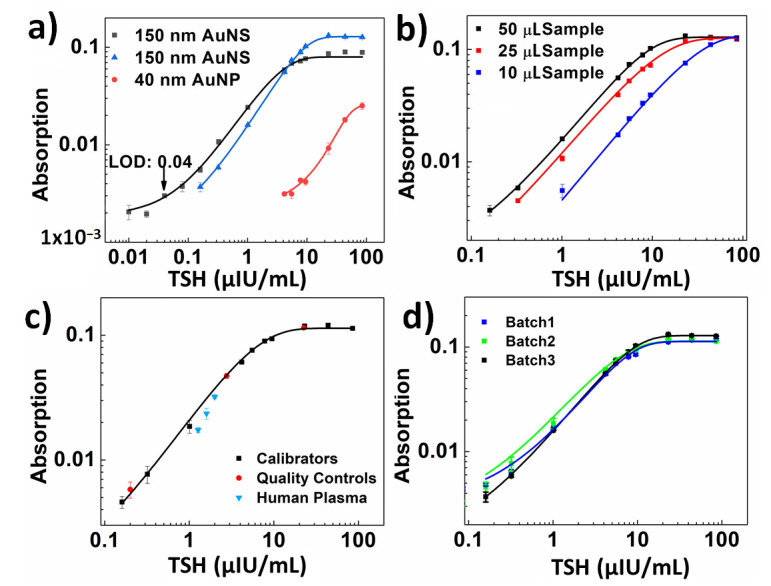
(**a**) Calibration curves for gold-nanoshell- and gold-nanoparticle-based LFAs with 50 µL of TSH calibrators. The calibration curve (black) obtained with gold nanoshells using DI water as blank showed an LOD of 0.04 µIU/mL. The calibration curve (blue) obtained with gold nanoshells using TSH-depleted serum as blank showed an LOD of 0.16 µIU/mL. (**b**) Calibration curves of gold-nanoshell-based LFAs with 10, 25, and 50 µL of TSH calibrators. (**c**) Calibration curve of gold-nanoshell-based LFA with 50 µL of TSH calibrators, quality controls, and human plasma samples. (**d**) Reproducibility of calibration curves on three different batches of TSH strips.

**Figure 4:**
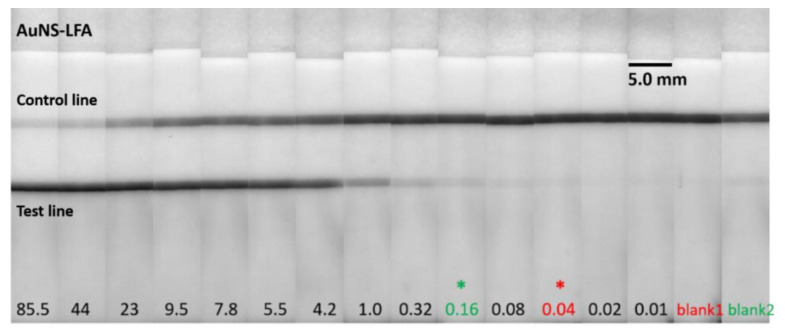
Representative photographs taken from the 150 nm gold-nanoshell-based LFAs with 50 µL sample volume. LOD of 0.04 µIU/mL was obtained when compared with blank 1 (DI water) and LOD of 0.16 µIU/mL was obtained when compared with blank 2 (TSH-depleted serum). The asterisks (∗) indicate the LOD.

In this study, LOD was assessed with DI water (blank 1) and TSH-depleted serum (blank 2) to investigate the effect of blank on the LOD. The calibration curves shown in Figure 3a were obtained by taking DI water and TSH-depleted serum as blank. They were fitted with the sigmoidal curve with dose-response function and are represented by Equations (1) and (2). A faint green color was observed on the LFA strip with TSH-depleted serum due to the low levels of TSH (Figure 4). The LFA strip with DI water showed no formation of the test line (Figure 4), indicating there was no non-specific binding involved in the assay, contributing to lower LOD. For the remainder of the study in determining LOD, TSH-depleted serum was used as a blank.

(1) $$\;y=-12.4+\frac{12.4}{\left(1+10^{\left(-15.2-x\right)*0.14}\right)}\;$$

(2) $$\;y=-0.15-\frac{0.026}{\left(1+10^{\left(-96-x\right)*0.09}\right)}$$

(3) $$\;\;y=-0.00124+\frac{0.0027}{\left(1+10^{\left(30.1-x\right)*0.027}\right)}$$

As mentioned earlier, AuNPs are commonly used in LFAs. Thus, an AuNS–LF test was compared with an AuNP–LF test under the same conditions and with the same antibodies and reagents. Comparing both the assays (Figure 3a), the AuNS–LF test showed an LOD of 0.16 µIU/mL, whereas the AuNP–LF test showed an LOD of 4.2 µIU/mL. The calibration curve obtained with AuNP is shown in Figure 3a. It was fitted with a sigmoidal curve with a dose-response function and is represented by Equation (3). In the case of AuNP–LF, the control line antibody did not show any intensity due to the poor brightness of the nanoparticles. These results demonstrate that AuNSs can enhance the sensitivity of conventional LFAs by 26 times, making it comparable to other sophisticated IVD techniques, such as enzyme-linked immunosorbent assay (ELISA). Such an improvement in sensitivity can be obtained by a simple change of the reporter nanoparticles without changing the geometry of the LFA device or adding any other incubation steps to further improve the sensitivity of the device.

**Figure 5:**
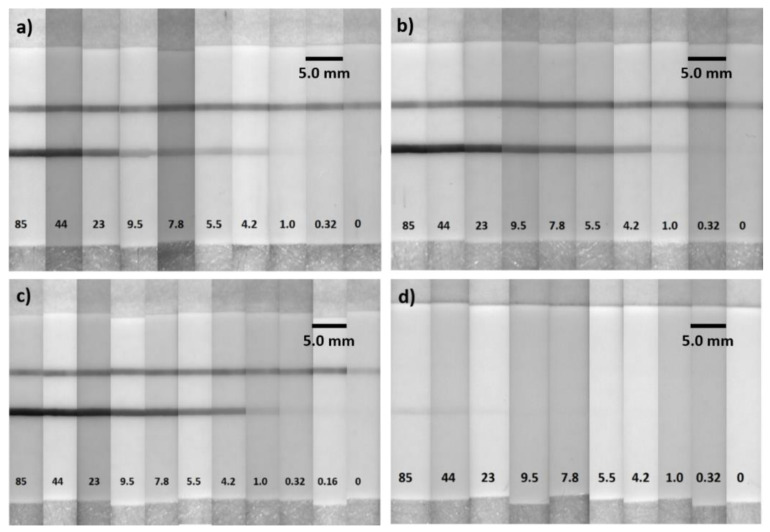
Representative photographs taken from the 150 nm gold-nanoshell-based LFAs with 10 (**a**), 25 (**b**), and 50 (**c**) µL of TSH calibrators. (**d**) Representative photographs taken from the 40 nm gold-nanoparticle-based LFAs with 50 µL of TSH calibrators.

To investigate the assay performance with respect to the sample volume, LFA experiments were performed with 10, 25, and 50 µL of TSH sample volume while maintaining a constant conjugate volume of 10 µL at 20 OD. The LFA strips were analyzed after 15 min for sample volumes of 10 and 25 µL. In the case of 50 µL sample volume, the LFA strips were analyzed after 30 min assay time. Figure 3b shows the calibration curves that correspond to 10, 25, and 50 µL of sample volume. We observed that the assay sensitivity improved with lower LOD and higher test line intensities as the sample volume increased. This was due to the excess of analyte available at higher sample volumes that was captured by all the detection antibodies linked to the AuNSs and by the capture antibodies at the test line, thereby preventing more AuNSs at the test line. Figure 3b shows that at 50 µL sample volume, the LOD was 0.16 µIU/mL, which was a 6.25-fold enhancement compared to the 10 µL sample volume and a 2.0-fold enhancement compared to the 25 µL sample volume. For the sample volume of 50 µL, the test line absorption was saturated at a concentration of over 23.1 µIU/mL. Thus, the dynamic range was determined to be 0.16–9.5 µIU/mL, as represented by Equations (1) and (2). For the sample volume of 25 µL, the absorption was saturated at a concentration above 43.9 µIU/mL, and the dynamic range was determined to be 0.32–23.1 µIU/mL, as represented by Equation (4). In the case of 10 µL sample volume, no saturation of absorption was observed, and the dynamic range was determined to be 1–43.9 µIU/mL, as represented by Equation (5). All the equations had an *r*-value of above 0.99. Figure 5a–c shows images of LFA strips after performing the assay with AuNS–TSH conjugate from 85.49 to 0.16 µIU/mL using 10, 25, and 50 µL of sample volume. Figure 5d shows the images of the LFA test strips that were obtained with AuNP–TSH conjugate at 50 µL sample volume.

(4) $$y=-0.95-\frac{0.822}{\left(1+10^{\left(-19.2-x\right)*0.045}\right)}\;\;\;$$

(5) $$y=-0.55-\frac{0.42}{\left(1+10^{\left(-34.4-x\right)*0.017}\right)}\;$$

Quality controls at three different levels of TSH and human plasma samples containing TSH at normal levels were used to assess the recovery of TSH and verify the developed assay. A recovery in the range of 70 to 130% was considered acceptable to validate the assay. Figure 3c shows the calibration curve along with quality controls and human plasma samples. In the case of quality controls at TSH levels of 22.6, 2.7, and 0.22 µIU/mL, the recovery was 100%, 115%, and 127%, respectively. For human plasma samples at TSH levels of 2.0, 1.6, and 1.27 µIU/mL, the recovery was 102%, 82.5%, and 72%, respectively. These data demonstrated that the performance of the AuNS–LFA strips was not affected by other biomarkers or by the complex matrix in human plasma, suggesting the feasibility of the AuNS–LFA strip in analyzing clinical samples. Figure S2 (Supplementary Materials) shows the images of LFA strips corresponding to the calibration curve in Figure 3c. The reproducibility of the TSH assay across three different batches of LFA strips was verified (Figure 3d). These data indicated that the results were reproducible from batch to batch of the TSH LFA strips prepared.

## 4. Conclusions

In this study, we developed a AuNS-based LFA for quantitative detection of serum TSH that can be used for diagnosis of hyperthyroidism (<0.5 µIU/mL) and hypothyroidism (>5.0 µIU/mL). The technique requires only 50 µL of sample volume and 30 min assay time. The developed TSH LFA shows high detection sensitivity and provides accurate quantitative analysis. The developed AuNS-based LFA strip does not require any additional incubation steps or device modifications to achieve high sensitivity of detection. The AuNS-based LFA test is ~25 times more sensitive than the conventional LFA test that utilizes 40 nm AuNPs as signal-generating labels. Combining all these merits, AuNS-based LFA can be considered among the most promising devices for TSH detection. In future, we aim to extend this work by developing an AuNS-based LFA strip for multiplexed detection of TSH and the thyroid hormones T3 and T4.

## Data Availability

The data that support the findings of this study are available from the corresponding author upon reasonable request.

## Supplementary Materials

The following supporting information can be downloaded at: www.mdpi.com/article/10.3390/bios12030182/s1. Figure S1: Shows the digital photograph of the lateral flow assay strip; Figure S2: Representative photographs taken from the 150 nm gold nanoshells-based LFAs with 50 µL of TSH calibrators, quality controls, and human plasma; Table S1: Shows the concentration of biomarkers in the calibrators.; Table S2: Shows the concentration of TSH in the disease state plasma as provided by the BIO-IVT and determined value by using the calibration curve; Table S3: Shows the concentration of TSH in the quality controls as provided by the Randox and determined value by using the calibration curve.

## References

[1] Fisher D.A. (1996). Physiological Variations in Thyroid Hormones: Physiological and Pathophysiological Considerations. Clin. Chem..

[2] Lin Z., Wang X., Li Z., Ren S., Chen G., Ying X., Lin J. (2008). Development of a Sensitive, Rapid, Biotin–streptavidin Based Chemiluminescent Enzyme Immunoassay for Human Thyroid Stimulating Hormone. Talanta.

[3] Jung W., Han J., Kai J., Lim J., Sul D., Ahn C.H. (2013). An Innovative Sample-to-Answer Polymer Lab-on-a-Chip with on-Chip Reservoirs for the POCT of Thyroid Stimulating Hormone (TSH). Lab Chip.

[4] Moreno M., de Lange P., Lombardi A., Silvestri E., Lanni A., Goglia F. (2008). Metabolic Effects of Thyroid Hormone Derivatives. Thyroid.

[5] Scanlan T.S., Suchland K.L., Hart M.E., Chiellini G., Huang Y., Kruzich P.J., Frascarelli S., Crossley D.A., Bunzow J.R., Ronca-Testoni S. (2004). 3-Iodothyronamine is an Endogenous and Rapid-Acting Derivative of Thyroid Hormone. Nat. Med..

[6] Weatherman R.V. (2007). A Triple Play for Thyroid Hormone. ACS Chem. Biol..

[7] Liu Y., Zhang Q., Wang H., Yuan Y., Chai Y., Yuan R. (2015). An Electrochemiluminescence Immunosensor for Thyroid Stimulating Hormone Based on Polyamidoamine-Norfloxacin Functionalized Pd–Au Core–shell Hexoctahedrons as Signal Enhancers. Biosens. Bioelectron..

[8] Leung A.M. (2015). Subclinical Hyperthyroidism is Associated with Increased Risks of Hip Fractures, Fractures at any Site, Nonspine Fractures, and Clinical Spine Fractures in the Largest Meta-Analysis to Date. Clin. Thyroidol..

[9] Taylor P.N., Albrecht D., Scholz A., Gutierrez-Buey G., Lazarus J.H., Dayan C.M., Okosieme O.E. (2018). Global Epidemiology of Hyperthyroidism and Hypothyroidism. Nat. Rev. Endocrinol..

[10] Garmendia Madariaga A., Santos Palacios S., Guillén-Grima F., Galofré J.C. (2014). The Incidence and Prevalence of Thyroid Dysfunction in Europe: A Meta-Analysis. J. Clin. Endocrinol. Metab..

[11] Vanderpump M.P.J. (2011). The Epidemiology of Thyroid Disease. Br. Med. Bull..

[12] Choi S., Hwang J., Lee S., Lim D.W., Joo H., Choo J. (2017). Quantitative Analysis of Thyroid-Stimulating Hormone (TSH) using SERS-Based Lateral Flow Immunoassay. Sens. Actuators B Chem..

[13] You D.J., Park T.S., Yoon J. (2013). Cell-Phone-Based Measurement of TSH using Mie Scatter Optimized Lateral Flow Assays. Biosens. Bioelectron..

[14] Ridgway E.C., McDermott M.T. (2001). Subclinical Hypothyroidism Is Mild Thyroid Failure and Should Be Treated. J. Clin. Endocrinol. Metab..

[15] Beitollahi H., Ivari S.G., Torkzadeh-Mahani M. (2018). Application of Antibody–Nanogold–Ionic Liquid–Carbon Paste Electrode for Sensitive Electrochemical Immunoassay of Thyroid-Stimulating Hormone. Biosens. Bioelectron..

[16] Pomelova V.G., Osin N.S., Bychenkova T.A., Paramonov D.V., Kostryukova T.S. (2017). Application of Eu(III) Nanoparticle Labels in Time-Resolved Phosphorescence Analysis for Detection of Thyroid Stimulating Hormone. Russ. J. Bioorg. Chem..

[17] Owen W.E., Gantzer M.L., Lyons J.M., Rockwood A.L., Roberts W.L. (2011). Functional Sensitivity of Seven Automated Thyroid Stimulating Hormone Immunoassays. Clin. Chim. Acta.

[18] Parolo C., Merkoçi A. (2013). Paper-Based Nanobiosensors for Diagnostics. Chem. Soc. Rev..

[19] Bahadır E.B., Sezgintürk M.K. (2016). Lateral Flow Assays: Principles, Designs and Labels. TrAC Trends Anal. Chem..

[20] Martinez A.W., Phillips S.T., Carrilho E., Thomas S.W., Sindi H., Whitesides G.M. (2008). Simple Telemedicine for Developing Regions: Camera Phones and Paper-Based Microfluidic Devices for Real-Time, Off-Site Diagnosis. Anal. Chem..

[21] Brangel P., Sobarzo A., Parolo C., Miller B.S., Howes P.D., Gelkop S., Lutwama J.J., Dye J.M., McKendry R.A., Lobel L. (2018). A Serological Point-of-Care Test for the Detection of IgG Antibodies Against Ebola Virus in Human Survivors. ACS Nano.

[22] Posthuma-Trumpie G.A., Korf J., van Amerongen A. (2008). Lateral Flow (Immuno)Assay: Its Strengths, Weaknesses, Opportunities and Threats. A Literature Survey. Anal. Bioanal. Chem..

[23] Qriouet Z., Cherrah Y., Sefrioui H., Qmichou Z. (2021). Monoclonal Antibodies Application in Lateral Flow Immunochromatographic Assays for Drugs of Abuse Detection. Molecules.

[24] Martinelli F., Scalenghe R., Davino S., Panno S., Scuderi G., Ruisi P., Villa P., Stroppiana D., Boschetti M., Goulart L.R. (2015). Advanced Methods of Plant Disease Detection. A Review. Agron. Sustain. Dev..

[25] Ahmed F.E. (2002). Detection of Genetically Modified Organisms in Foods. Trends Biotechnol..

[26] Luo K., Kim H., Oh M., Kim Y. (2020). Paper-Based Lateral Flow Strip Assay for the Detection of Foodborne Pathogens: Principles, Applications, Technological Challenges and Opportunities. Crit. Rev. Food Sci. Nutr..

[27] Yao L., Xu J., Cheng J., Yao B., Zheng L., Liu G., Chen W. (2022). Simultaneous and Accurate Screening of Multiple Genetically Modified Organism (GMO) Components in Food on the Same Test Line of SERS-Integrated Lateral Flow Strip. Food Chem..

[28] Bergua J.F., Hu L., Fuentes-Chust C., Álvarez-Diduk R., Hassan A.H.A., Parolo C., Merkoçi A. (2021). Lateral Flow Device for Water Fecal Pollution Assessment: From Troubleshooting of its Microfluidics using Bioluminescence to Colorimetric Monitoring of Generic *Escherichia coli*. Lab Chip.

[29] Grubb A.O., Glad U.C. (1979). Immunoassay with Test Strip Having Antibodies Bound Thereto. U.S. Patent.

[30] Gasperino D., Baughman T., Hsieh H.V., Bell D., Weigl B.H. (2018). Improving Lateral Flow Assay Performance using Computational Modeling. Annu. Rev. Anal. Chem..

[31] Zhou W., Gao X., Liu D., Chen X. (2015). Gold Nanoparticles for In Vitro Diagnostics. Chem. Rev..

[32] Huang X., El-Sayed M.A. (2010). Gold Nanoparticles: Optical Properties and Implementations in Cancer Diagnosis and Photothermal Therapy. J. Adv. Res..

[33] Mayer K.M., Hafner J.H. (2011). Localized Surface Plasmon Resonance Sensors. Chem. Rev..

[34] Willets K.A., Van Duyne R.P. (2007). Localized Surface Plasmon Resonance Spectroscopy and Sensing. Annu. Rev. Phys. Chem..

[35] Eustis S., El-Sayed M. (2006). Why Gold Nanoparticles are More Precious than Pretty Gold: Noble Metal Surface Plasmon Resonance and its Enhancement of the Radiative and Nonradiative Properties of Nanocrystals of Different Shapes. Chem. Soc. Rev..

[36] Cao-Milán R., Liz-Marzán L.M. (2014). Gold Nanoparticle Conjugates: Recent Advances toward Clinical Applications. Expert Opin. Drug Deliv..

[37] Kumar S., Aaron J., Sokolov K. (2008). Directional Conjugation of Antibodies to Nanoparticles for Synthesis of Multiplexed Optical Contrast Agents with both Delivery and Targeting Moieties. Nat. Protoc..

[38] Love J.C., Estroff L.A., Kriebel J.K., Nuzzo R.G., Whitesides G.M. (2005). Self-Assembled Monolayers of Thiolates on Metals as a Form of Nanotechnology. Chem. Rev..

[39] Wang Y., Rhéaume É., Lesage F., Kakkar A. (2018). Synthetic Methodologies to Gold Nanoshells: An Overview. Molecules.

[40] Liu Y., Zhan L., Qin Z., Sackrison J., Bischof J.C. (2021). Ultrasensitive and Highly Specific Lateral Flow Assays for Point-of-Care Diagnosis. ACS Nano.

[41] Wu L., Qu X. (2015). Cancer Biomarker Detection: Recent Achievements and Challenges. Chem. Soc. Rev..

[42] Perfézou M., Turner A., Merkoçi A. (2012). Cancer Detection using Nanoparticle-Based Sensors. Chem. Soc. Rev..

[43] Qin Z., Chan W.C.W., Boulware D.R., Akkin T., Butler E.K., Bischof J.C. (2012). Significantly Improved Analytical Sensitivity of Lateral Flow Immunoassays by using Thermal Contrast. Angew. Chem. Int. Ed..

[44] Yang Y., Ozsoz M., Liu G. (2017). Gold Nanocage-Based Lateral Flow Immunoassay for Immunoglobulin G. Microchim. Acta.

[45] Loynachan C.N., Thomas M.R., Gray E.R., Richards D.A., Kim J., Miller B.S., Brookes J.C., Agarwal S., Chudasama V., McKendry R.A. (2018). Platinum Nanocatalyst Amplification: Redefining the Gold Standard for Lateral Flow Immunoassays with Ultrabroad Dynamic Range. ACS Nano.

[46] Gao Z., Ye H., Tang D., Tao J., Habibi S., Minerick A., Tang D., Xia X. (2017). Platinum-Decorated Gold Nanoparticles with Dual Functionalities for Ultrasensitive Colorimetric In Vitro Diagnostics. Nano Lett..

[47] Yen C., de Puig H., Tam J.O., Gómez-Márquez J., Bosch I., Hamad-Schifferli K., Gehrke L. (2015). Multicolored Silver Nanoparticles for Multiplexed Disease Diagnostics: Distinguishing Dengue, Yellow Fever, and Ebola Viruses. Lab Chip.

[48] Rayev M., Shmagel K. (2008). Carbon–Protein Covalent Conjugates in Non-Instrumental Immunodiagnostic Systems. J. Immunol. Methods.

[49] Linares E.M., Kubota L.T., Michaelis J., Thalhammer S. (2012). Enhancement of the Detection Limit for Lateral Flow Immunoassays: Evaluation and Comparison of Bioconjugates. J. Immunol. Methods.

[50] Juntunen E., Myyryläinen T., Salminen T., Soukka T., Pettersson K. (2012). Performance of Fluorescent Europium(III) Nanoparticles and Colloidal Gold Reporters in Lateral Flow Bioaffinity Assay. Anal. Biochem..

[51] Wang Y., Fill C., Nugen S.R. (2012). Development of Chemiluminescent Lateral Flow Assay for the Detection of Nucleic Acids. Biosensors.

[52] Sakurai A., Takayama K., Nomura N., Yamamoto N., Sakoda Y., Kobayashi Y., Kida H., Shibasaki F. (2014). Multi-Colored Immunochromatography using Nanobeads for Rapid and Sensitive Typing of Seasonal Influenza Viruses. J. Virol. Methods.

[53] Badu-Tawiah A., Lathwal S., Kaastrup K., Al-Sayah M., Christodouleas D.C., Smith B.S., Whitesides G.M., Sikes H.D. (2015). Polymerization-Based Signal Amplification for Paper-Based Immunoassays. Lab Chip.

[54] Tam J.O., de Puig H., Yen C., Bosch I., Gómez-Márquez J., Clavet C., Hamad-Schifferli K., Gehrke L. (2017). A Comparison of Nanoparticle-Antibody Conjugation Strategies in Sandwich Immunoassays. J. Immunoass. Immunochem..

[55] Tuersun P., Yusufu T., Yimiti A., Sidike A. (2017). Refractive index sensitivity analysis of gold nanoparticles. Optik.

[56] Tuersun P. (2015). Optimizing the figure of merit of gold nanoshell-based refractive index sensing. Optik.

[57] Omrani M., Mohammadi H., Fallah H. (2021). Ultrahigh sensitive refractive index nanosensors based on nanoshells, nanocages and nanoframes: Efects of plasmon hybridization and restoring force. Sci. Rep..

[58] Chen H., Kou X., Yang Z., Ni W., Wang J. (2008). Shape- and size-dependent refractive index sensitivity of gold nanoparticles. Langmuir.

