# Supplementary material for: High-Sensitive Detection and Quantitative Analysis of Thyroid-Stimulating Hormone Using Gold-Nanoshell-Based Lateral Flow Immunoassay Device
Source: Biosensors (Basel). 2022 Mar 19;12(3):182. doi: 10.3390/bios12030182 (PMC8946628; doi:10.3390/bios12030182)
Supplement: Supplementary file 1 [file biosensors-12-00182-s001.zip › biosensors-1562977-supplementary.pdf]

Supplementary Materials

Table S1: Shows the concentration of biomarkers in the calibrators.

Calibrator	TSH (µIU/ml)	FT3 (ng/ml)	FT4 (ng/ml)
1	0.08	0.11	0
2	0.16	0.23	0
3	0.327	0.46	0
4	1.014	1.4	1.27
5	4.182	3.4	3.38
6	5.54	7.47	7.61
7	7.839	12.48	10.25
8	9.501	25.05	17.29
9	23.177	58.03	35.49
10	43.903	96.2	47.89
11	85.49	193.44	138.02

Table S2: Shows the concentration of TSH in the disease state plasma as provided by the BIO-IVT and determined value by using the calibration curve.

Plasma Samples	TSH levels (µIU/ml) as given by Bio-IVT	TSH levels(µIU/ml) determined	Recovery (%)
74343A1	1.27	0.92	72
74388A1	1.6	1.32	82.5
406430A2	2	2.05	102

Table S3: Shows the concentration of TSH in the quality controls as provided by the Randox and determined value by using the calibration curve.

Quality Controls	TSH levels (µIU/ml) as determined by Roche Elecsys	TSH levels(µIU/ml) determined	Recovery (%)
L3	22.6	22.7	100
L2	2.7	3.13	115
L1	0.22	0.27	127

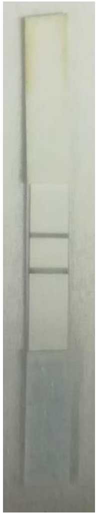

Figure S1: Shows the digital photograph of the lateral flow assay strip.

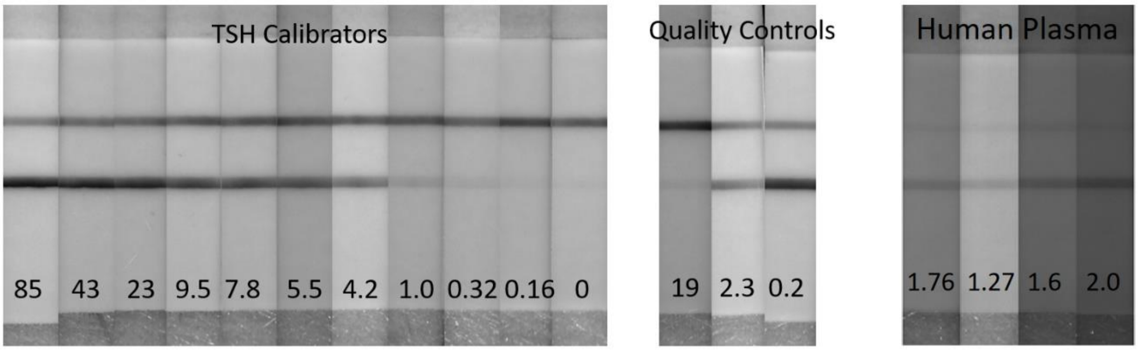

Figure S2: Representative photographs taken from the 150 nm gold nanoshells-based LFAs with 50 µl of TSH calibrators, quality controls, and human plasma.
